# Annotations

**Published:** 1891-10-10

**Authors:** 


### Annotations.

Mr. T. Bayley, one of the Parliamentary candidates for Chesterfield, speaking at an open-air demonstration in aid of the local hospital, said he "did not think that the religious denominations had done quite so much as they ought for the medical charities. He believed that there was very often more Christianity taught in the hospital on Sunday than in some of our places of worship. He wished men and women would indulge less in the argument of the pulpit and do more Christian work." This good work of hospitals is an aspect of Christian virtue which is often forgotten. The same Parliamentary candidate said he wished there was less preaching. We do not agree with him. In our judgment, if there were less preaching there would be less practice and less philanthropy. If he had demanded better preaching, and preaching of which the burden was practice, we should have given him unqualified approval. It cannot, however, be doubted that hospitals are important centres of religious education. Both in motive and in method, most hospitals are religious from beginning to end. What the patient sees as he lies in bed from day to day is discipline, duty, self-denial, and charity in some of their highest forms. This is the practical aspect of Christianity; the aspect which appeals most powerfully to the poor, the suffering, and the ignorant. If the voluntary hospitals were taken away, one of the most convincing of all the Christian evidences would be removed with them. So long as those hospitals stand in their places and prosper, so long will Christendom bid defiance to some of the most formidable attacks that are made upon it. The hospital, undoubtedly, is in every town a bulwark of the Church. But if the Hospital, as a form of practical religion, is a bulwark of the Church, the Church in its turn, as a teacher of the first principles of religion, must take care always to give the Hospitals a place in the very forefront of the argument.

**Hospital Religion.**

Under the title of "Our Unseen Foes, and How to Meet Them," Mr. A. Wheeler publishes an interesting little book. On the cover he prints the names Koch, Pasteur, and Lister; and the unwary might imagine that those famous personages were the unseen foes which Mr. Wheeler is anxious to teach us how to meet. Bacteria, however, and not bacteriologists, are our real invisible adversaries. In the present state of knowledge, it is not easy to write a book about bacteria for popular readers which shall be of great service to them. Mr. Wheeler has, however, carried out his task with much skill; and has, at least, made a readable and interesting story. The bacteriologist had need be a skilful and discriminating person, since he has tasks to perform of the very nicest description. All of us have been familiar from childhood with moulds and fungi; but we have not been aware of the peculiar part played by certain varieties of similar minute organisms in the story of the diseases of plants, animals, and man. The bacteriologist has entered upon a practically new region of scientific enquiry. It will take him probably many years to discover for us all we want to know about these small living forms, and probably a much longer period before he can teach us how to deal with them so as to render them all equally harmless. It is with bacteria as it is with the cooking of a hare: you must first catch your hare and your bacteria. The bacteriologist does this by cultivations, and nature shows him the way. When nature wants to grow new moulds she brings about a period of damp weather, and exposes all sorts of nutrient materials, which tempt the organisms to settle down and commence the work of multiplication. A piece of meat, raw or cooked, a bowl of stock kept a little too long, a pair of old boots, or an unprotected jam pot; any of these will serve her immediate purpose. The bacteriologist, having once got his cue from nature, can easily cultivate all the available varieties of bacteria by means similar to those which nature employs. But cultivation is not everything: differentiation is equally essential. Bacteria must be separated into classes like potatoes, green peas, and human beings; and the little organisms lend themselves to differentiation by their peculiar appetites for different stains or dyes. When we have caught our bacteria, and separated them, we want to know which are safe and which are dangerous, and not only so, but under what circumstances the dangerous ones are most injurious. What we want to know still more is how to render the injurious ones harmless; and if that be impossible, how to put an end to their lives. We want to know more intelligently also the peculiar conditions of the human organism which render it especially liable to bacterial attacks. A great many facts on all these points are told us by Mr. Wheeler in his little book. Whether these facts will be of any great service to the general public at the present time we are not prepared to say; but that the book is clearly written and very readable we unhesitatingly affirm. The publishers are Messrs. John Wright and Co., of Bristol.

**Our Unseen Foes.**

The Folk-lore people are true men of science, although, to use an Irishism, most of them are women. They study the most interesting of all sciences, the science of man; and they study it in the man pure and simple, that is in the common man, not in the philosopher. The philosopher is a tremendous animal to tackle. One never knows whether he is an eel or a sea serpent. What one does know is that he is the slipperiest of the slippery, and can wriggle amazingly. The Folk-lore people, with fine discrimination, give him a wide berth. The little Congress, over which Mr. Andrew Lang has presided with his characteristic sweetness, has proved a great comfort to many minds. It is always consoling to the uninitiated to find how very little the initiated really know about the mysteries whose hierarchs they profess to be. Whence came the thousand and one myths and fairy tales, legends, and historical traditions which float about like ghostly bacteria in the atmosphere of every corner of the earth? Did they spring up in one common centre, or in two or three principal centres, and were they thence disseminated by adventurous travellers all over the world as thistle-down is carried by the various winds? Or are the roots of all such myths and legends inherent in the common mind of humanity, and have they originated more or less simultaneously in every part of the globe as flies seem to be evolved wherever there is moisture, warmth, and sunshine? To these knotty questions the Folk-lorists devoted themselves with all due seriousness, and, like true men of science, their opinions have proved to be not only many and diverse, but in some cases adverse and opposite, and mutually excludatory. Happily there was no fighting; the Folk-lorists are people of good taste, and of mild and genial disposition. It does not really much matter whether the story of Cinderella, and of the forty thieves, originated in a common centre, and were thence widely disseminated, or sprang up more or less simultaneously at Pekin, Colorado, and John o' Groat's House: and, to do them justice, the Folk-lorists in Congress seemed as if they did not particularly care. One thing is to be learnt from the Congress; and that is that exact knowledge of remote history is exceedingly difficult to obtain. Perhaps also we may take to heart the fact that Congressional meetings of learned and enquiring folk can be both pleasant and profitable, alike to themselves and to the world, without annoying anybody, or making even the most insignificant person feel his insignificance. Congresses of all kinds, even when they do not elicit any new truth, are useful for the diffusion of reasonable opinions and kindly feeling. May they continue to prosper so long as they continue to carry on their operations sweetly and reasonably.

